# *Announcement*: Childhood Cancer Awareness Month — September 2017

**DOI:** 10.15585/mmwr.mm6636a7

**Published:** 2017-09-15

September marks Childhood Cancer Awareness Month. Each year, approximately 15,000 U.S. children and adolescents aged <20 years receive a cancer diagnosis; leukemia, brain tumors, and lymphoma are the most common types of cancers that affect this age group (*1*). During the past 4 decades, largely because of advances in the efficacy of treatment and supportive care, 5-year relative survival for childhood cancers increased from 62% to 85% (*2*). However, for some childhood cancers, such as brain or bone tumors, 5-year relative survival remains <75% (*2*). In addition, child and adolescent cancer survivors often face long-term complications, including heart disease, infertility, or secondary cancers related to their treatment, and need lifelong survivorship care planning (*3*).

CDC addresses the needs of children and adolescents living with, through, and beyond cancer by collecting and analyzing data and using scientific knowledge to develop and implement interventions. CDC works with local, state, and national partners to address disparities in referral to, enrollment in, and availability of childhood cancer clinical trials. To strengthen cancer survivorship care for children and adolescents, CDC collaborates with partner agencies to research barriers to clinical trial enrollment and interventions to improve care planning and self-management after completion of cancer treatment.

United States Cancer Statistics surveillance data (https://nccd.cdc.gov/uscs/) are important for monitoring childhood cancer incidence and mortality. CDC’s Pediatric and Young Adult Early Case Capture program (https://www.cdc.gov/cancer/npcr/early-case-capture.htm) specializes in rapid reporting of childhood cancer data, which can provide clinicians, researchers, and public health professionals with timely, relevant data. Additional information is available at https://www.cdc.gov/cancer/.
